# Health system bottlenecks hindering provision of supportive and dignified maternity care in public health facilities

**DOI:** 10.1371/journal.pgph.0000550

**Published:** 2022-07-08

**Authors:** Waqas Hameed, Bushra Khan, Sameen Siddiqi, Muhammad Asim, Bilal Iqbal Avan

**Affiliations:** 1 Department of Community Health Sciences, Aga Khan University, Karachi, Pakistan; 2 Department of Psychology, University of Karachi, Karachi, Pakistan; 3 Department of Clinical Research, London School of Hygiene and Tropical Medicine, London, United Kingdom; Liverpool School of Tropical Medicine, UNITED KINGDOM

## Abstract

Mistreatment with women during childbirth is prevalent in many in low- and middle-income countries. There is dearth of evidence that informs development of health system interventions to promote supportive and respectful maternity care in facility-based settings. We examined health systems bottlenecks that impedes provision of supportive and respectful maternity care in secondary-level public healthcare system of Pakistan. Using a qualitative exploratory design, forty in-depth interviews conducted with maternity care staff of six public health facilities in southern Pakistan. Development of interview guide and data analyses were guided by the WHO’s six health system building blocks. A combination of inductive and deductive approach was used for data analyses. Our study identified range of bottlenecks impeding provision of RMC. In terms of leadership/governance, there was lack of institutional guidelines, supervision and monitoring, and patient feedback mechanism. No systematic mechanism existed to screen and record patient psychosocial needs. Health workforce lacked training opportunities on RMC that resulted in limited knowledge and skills; there were also concerns about lack of recognition from leadership for good performers, and poor relationship and coordination between clinical and non-clinical staff. Regarding the domain of service delivery, we found that patients were perceived as un-cooperative, non-RMC manifestations were acceptable and normalized under certain conditions, and restrictive policies for active engagement of companions. Finally, lack of cleanliness, curtains for privacy, seating arrangement for companion were the identified issues infrastructural issues. A service-delivery intervention package is needed that effectively uses all six components of the health system: from investments in capacity building of maternity teams to creating a conducive facility environment via proper governance and accountability mechanisms. Such interventions should not only focus on provision of maternity care in a respectful and dignified manner, but also ensure that care is responsive to the psychosocial needs of pregnant women without any discrimination.

## Introduction

In the year 2014, the World Health Organization (WHO) issued an affirmative policy directive to promote respectful maternity care (RMC), stating that “every woman has the right to the highest attainable standard of health, which includes the right to dignified, respectful health care” [1]. The same is also reflected in WHO’s vision for improved quality of maternal and newborn care that highlights three domains influencing positive women’s experience: respect and dignity, effective communication, and emotional support [2]. More recently, a comprehensive set of evidence-based recommendations were published by the WHO which aim at promoting positive user experience of intrapartum care [3]. These guidelines defined RMC as a combined construct of supportive and respectful care stating that “the care organised for and provided to all women in a manner that maintains their dignity, privacy and confidentiality, ensures freedom from harm and mistreatment, and enables informed choice and continuous support during labour and childbirth” [3].

Despite the growing recognition that mistreatment during childbirth is a violation of women’s human rights [4], research conducted over the past decades provide clear evidence of demeaning intrapartum care in health facilities worldwide, particularly in low- and middle-income countries (LMICs) [5–10]. Mistreatment may lead to short- and long-term adverse consequences such as pain and suffering, negative birthing experiences, fear of childbirth, and feeling of dehumanisation [7,11]. Such woeful experiences may serve as a deterrent for facility-based births [5,12,13]. Given that women are generally the victim of mistreatment during childbirth, largely (and understandably) the focus of research has been around women’s perspective of care [5,6,8–10,14]. In contrast, fewer studies have been conducted that explore the insights of service providers regarding the issue [15–17]. Of these enquiries, some focused on perceptions of service providers regarding mistreatment [18] and its potential impact on the well-being of pregnant women [17]; while others have also explored individual and systemic drivers of mistreatment [16,19] and how broader socio-cultural norms influence mistreatment [20].

These qualitative studies with service providers have identified several interlinked underlying factors of mistreatment that can broadly be classified into: individual level factors (provider attitude, e.g., lack of perceived benefit of RMC), gaining compliance to ensure good outcomes, perception of women being difficult, stress and burnout); systemic factors (workload, lack of accountability, facility culture, lack of medicines and supplies); and socio-cultural factors (power dynamic between patient and providers, normalised behaviour for punishing “disobedient” women, gender norms) [15–17,20,21]. While these studies contribute significantly to the current body of knowledge on the issue, most have explored the perspectives of service providers using the framework of mistreatment proposed by Bowser et al. [12] and Bohren et al. [5], and few have also studied it through the lens of behavioural science [22–24]. However, to our knowledge, there is limited evidence that the underlying challenges of mistreatment from a health systems perspective have been systematically examined. Moreover, these investigations do not comprehensively address specific health system issues (especially inputs and processes) that surround implementation of RMC.

In our view, investigation of this issue has its own importance for reasons that: a) RMC is considered as an integral part of high-quality healthcare [25], hence any deviance from such care is fundamentally characterised as a problem in health system’s responsiveness [26,27]; b) the tested RMC interventions are deemed promising but lack sustainability of demonstrated effect [28] due to the concerns around how well these interventions are embedded within healthcare system [28]; and c) evidence also show unidimensional intervention (e.g. capacity building of service providers) may not necessarily lead to any improvement in RMC [29] as health system challenges may limit the translation of providers’ positive attitude and behaviours into practice of RMC [30]. Therefore, improvements in RMC will remain a challenge unless the issue is viewed through a health systems lens that address the bottlenecks in the provision of RMC [28,29].

By and large, there is limited evidence around health system bottlenecks that hinder provision of respectful maternity care in public health systems of LMICs. Additionally, most of the researches that gathered service providers’ perspectives on RMC have been conducted in African countries [15–17,20,21], leaving an evidence gap from South Asian countries. To fill this knowledge gap, we aimed to describe how a typical maternity ward and labour room services operate within an Obstetrics and Gynaecology section of public health facility, and describe systemic bottlenecks that impede provision of respectful maternity care in secondary-level healthcare facilities in Pakistan.

### Country context

With a population of over 225 million inhabitants [31], Pakistan has one of highest rates of maternal mortality [32], and is ranked the riskiest country for newborns [33]. Half of the female population is illiterate [34] and the country ranked 153 out of 156 in terms of gender gap [35]. The healthcare in Pakistan is delivered through a three-tiered health system: primary, secondary and tertiary. This research focused on secondary-level healthcare where inpatient childbirth facilities are formally introduced through maternity ward and labour room services. The training curriculum of midwifery do cover topics around ethics, respect and dignity of patient; however, these learning are not probably translated into practice as indicated in the recent study from secondary-level health facilities which revealed high reports of disrespectful and abusive intrapartum care [14]. This in turn leads to a startlingly low (22%) proportion of total births that take place in public health facilities [34]. To the best of authors’ knowledge, RMC related content has been included occasional trainings conducted by NGOs; however, no formal intervention has been developed and tested to promote RMC in Pakistan.

## Methods

### Study design and settings

This qualitative enquiry was part of a larger study that aims to develop and test feasibility of a service-delivery intervention model to promote the culture of support and respect during childbirth in public health facilities [36]. A qualitative exploratory design was adopted using in-depth interview technique. Six secondary-level public health facilities providing at least basic emergency obstetric and newborn care (BEmONC) services were selected from Thatta and Sujawal districts of Southern Sindh (three facilities in each district). These contiguous districts have a population of around 0.8 million [37] each and are approximately 95 kilometres away from Karachi, the most populous metropolitan city of Pakistan.

### Recruitment of study participants

Three groups of participants were identified for this study: (1) clinical staff (obstetrician/gynaecologist, midwife, lady health visitor (LHV), nurse, technician; (2) non-clinical staff (*Aaya*, housekeeping, security guard); and (3) hospital managers (Medical Superintendent, Health Information System Officer). The inclusion of non-clinical staff is based on their involvement in childbirth processes to support clinical staff [38] in low resource settings. Health facility staff working in the maternity wards during the data collection period were eligible to participate in the study. IDIs were conducted to minimise the issue of courtesy bias and the concerns regarding breach of confidentiality through disclosure of poor practices. The study participants were selected purposively as per pre-defined quota according to cadre of service providers. We met with the head of maternity section who nominated relevant clinical and non-clinical members of maternity team based on the criteria the research team proposed to the head–for example, after interviewing an experienced nurse, the second nurse participant would be relatively less experienced and have slightly different responsibilities (e.g. labour room duty / ward duty / admission and discharge counter).

### Interview guide development

The primary intent of this study was to give directions for the development of health system intervention for promoting RMC. Hence, we used WHO’s framework of health system building blocks which encompasses all aspects of the health system [39]. This framework guided development of three separate semi-structured interview guidelines: clinical and non-clinical staff, and hospital managers (see S1 and S2 Text). Besides typical open-ended questions, participants were also given scenarios around different manifestations of mistreatment to get a deeper understanding of their beliefs and values by minimizing social-desirability bias. These scenarios were developed based on a literature review [20] and in consultation with practicing maternal health experts. Furthermore, we employed three tools of participatory reflection analysis (PRA)–Timeline, ‘*Chapati*’ (or Venn) diagram, and flow diagram [40] respectively to gather information about daily work routine of health staff, major challenges that surround RMC, and mechanisms to address challenges encountered during care provision. It is important to note that these PRA techniques were embedded within the interview guide–for example, the respondents were asked to explain their daily routine in a paper using paper and pen by drawing a line. These techniques were not separately administered to validate or substantiate the interview responses. The interview guide was piloted with a few maternity team members of a different public hospital.

### Data collection

A total of 40 in-depth interviews were conducted between February and June 2020. The duration got prolonged due to the suspension of field activities from March–May 2020 because of COVID19 pandemic. Interviews were conducted by a team of two trained female sociologists, who further underwent a three-day training prior to commencement of data collection. All interviews were conducted in local Sindhi language at health facilities except for 4 which took place at participants’ home and all were audio recorded with the permission of study participants. On average each interview lasted approximately 45 to 60 mins. No personal identification was linked to the data.

### Data analysis

We used a combination of inductive and deductive approaches for data analyses. Initial codes were guided by pre-existing themes in the interview guide and new categories were derived from the data. And finally, the two sets of categories were reduced according to WHO’s six health system building block framework in consultation with other co-investigators [39,41].

The audio-recordings were transcribed verbatim by the interviewers and translated into English by a linguistic expert. Two researchers (WH and MA) independently reviewed the transcriptions to perform coding. Transcripts were read multiple times to ascertain the true meaning of the participants’ responses. Initial categories of codes and the emerging categories were recorded separately, subsequently merged into main themes and aligned with the broader domains of WHO building blocks. The transcriptions were organised, reviewed, and analysed using NVivo version 11.0. The rigor in qualitative research was delineated by applying consolidated criteria for reporting qualitative research (COREQ) guideline [42]. This guideline ensured the credibility, conformability, dependability, and transferability of study findings. The credibility and reliability of the results were ensured using multiple approaches: a) the in-depth interviews were conducted by an independent newly hired team of data collectors, b) the data analyst discussed the interpretation of transcriptions with data collectors and sought consensus, and c) since the study was part of an intervention research that included training of maternity staff of same health facilities, the researchers shared and verified these study findings by the maternity staff during trainings [36].

### Ethical consideration

Ethical approval was obtained from the ethics review committee of the Aga Khan University (approval number: 2019-1683-5607) and the ethics committee of London School of Hygiene and Tropical Medicine (approval number: 17928). All participants gave written informed consent.

## Results

The main study findings are organised according to six core components of health systems building blocks. In order to support a better understanding of the results for the readers, a brief description on the routine operations of Obstetrics & Gynaecology section within a secondary-level public health facility is presented in S3 Text.

A total of 40 IDIs were included in the analysis. Table 1 shows the socio-demographic characteristics of participants. The mean age was 39 (±7.7) years and had an experience of 13 (±8.3) years. Nearly all were female (90%), 58% were clinical staff, and about 45% were working in a morning shift.

**Table 1:** Characteristics of study participants.

Characteristics	n (%)
**Sex**	
Female	36 (90)
Male	4 (10)
**Age in years**	
Mean (±SD)	39 (±7.7)
**Health worker cadre**	
Clinical staff	23 (58)
Non-clinical staff	15 (38)
Health managers	2 (5)
**Total experience in years**	
Mean (±SD)	13.4 (±8.3)
**Working shifts**	
Morning	18 (45)
Evening	12 (30)
Night	4 (10)
Rotation	6 (15)

### 1 Service delivery (services that are responsive to, individual patient preferences, needs and values, and provided in respectful manner)

#### 1.1 Interaction with patients and their companion

Uncooperative and demanding patients and their companions are considered as a biggest challenge for the staff. According to most of the clinical staff, dealing with patients and their companions with unreasonable demands such as provision of drip, medicines, immediate conduction of normal delivery upon arrival, provision of services that either the patient is not entitled of or are beyond the remit of health facility (e.g. blood transfusion, delivery of anaemic patient etc.) is a huge challenge. On the other hand, non-clinical staff expressed concerns about the carelessness of patients in maintaining cleanliness of health facilities.

***“Almost all the villagers come here who do not understand the situation*. *They say that ‘just do this’*, *whether we have the equipment or not to treat the patient”*. *Lady Health Visitor*, *Sujawa***l

While the both clinical and non-clinical staff do acknowledge that literacy is low among patients and companions, repeated counselling and continuous motivation due to this low health literacy was still perceived a challenge. When the expectations of patients are not immediately met or the staff fails to effectively explain the reasons behind it, they patients or their companions become intolerant and aggressive and they use rude and abusive language, or made baseless allegation on staff which is deemed difficult to deal with by the staff.

“***People do physical violence with our staff*. *Once female patient was expired in the morning*, *her family members broke the glasses of windows”*. *Nurse*, *Thatta***

“***The companions create a lot of mess in the wards*. *They eat and throw the trash on the floor*. *They don’t understand*, *no matter how many times you explain it to them”*. *Sweeper*, *Sujawal***

#### 1.2 Provision of respectful maternity care

There is a perception among clinical staff that being strict with patients can ensure adherence to the instructions especially at the time of childbirth leads to verbal or physical abuse with the intention to save life of a newborn and mother. Few clinical and non-clinical staff also linked such behaviour with individual personality traits–as those who are short-tempered naturally misbehave with everyone including patients and their co-workers without any particular reasons.

“***When baby’s head starts appearing then women close their legs*. *Baby stuck in the middle*. *Women need a slap because without it they don’t cooperate*.*” Aaya*, *Thatta***

“***Staff nurses misbehave with patients tell them bad things like didn’t you feel the pain in the wedding night that you are shouting now with the pain” Cleaner*, *Thatta***

“***Some doctors think that Poor people never complain even if you or disrespect them”*. *Lady health visitor*, *Sujawal***

Another common underlying reason for non-respectful care is persistent unreasonable demands from patients (described above) that are beyond the remit of health facility as it creates tension between patients and healthcare providers. Patients with unmet demands tend to make baseless allegations on service providers hence service providers sometime retaliate which leads to disrespect behaviours. An impression among patient that staff is deliberately not giving the services while according to staff the health facility is not equipped with these services. This creates confusion and tension between patients and staff and hence it leads to disrespect and abuse. According to both clinical and non-clinical staff, non-respectful care is a reaction of rude behaviour of patients. Regarding neglect and discrimination, overworked and socio-political influence respectively were the cited as the common reasons. For example, patients who have strong affiliations will influence the staff for getting immediate care by making phone calls from an influential persons of the area that leads to discrimination with other patients. At the district headquarter hospital (bigger hospitals), overworked of the staff member constrained their ability to pay optimum attention to the patients as desired.

“***People force us to give them treatment here even if we don’t have the services available here*. *They show stubbornness that why government has built such a big hospital”*. *Lady Health Visitor*, *Thatta***

“***Many people create fuss*. *Our behaviour depends on their behaviour”*. *Operation Theatre Technician*, *Thatta***

Husbands are not allowed in Obs and Gyne section and staff also reported a feeling of uncomfortableness in their presence at the time of delivery. Female companions are usually not allowed in the labour room due to confidentiality issue as there are multiple beds in a labour room without separators. Generally, companions are engaged on needs basis such as issue of language barrier, women is under immense stress, non-compliant or if she has some form of disability.

“***In my opinion every woman has certain level of privacy*. *Even her husband should not see her in that state*. *Plus*, *I and my helper are also there*. *He is her husband not ours*. *Woman is nude in front of a man and I am also standing there*, *I won’t allow this”*. *Midwife*, *Thatta***

“***Companion usually cooperate*, *especially for dumb and deaf patients come so we invite their companion in labour room”*. *Gynaecologist*, *Sujawal***

#### 1.3 Identifying differential needs and preferences of patients

When asked about how staff identifies different needs of patient, majority of them describes that patient needs are distinguished based on clinical grounds–like the stage of labour, blood pressure, blood deficiency, labour pain, fetal movement, potential complications etc.

“***Every patient has different expectation and needs*. *If patient is very weak or anaemic*, *then we refer her to the big hospitals*. *If they are mentally stressed*, *then we do their counselling”*. *Midwife*, *Thatta***

In terms of non-clinical factors, poverty was mainly identified as the main characteristic by both clinical and non-clinical staff. Participants also identified other factors such as unfamiliarity with native language, physical disability, and stress. Mostly referred stress to as being ‘upset’ for various reasons; however, no one linked it with known common mental conditions such as anxiety or depression. Staff, at times, provide money out-of-pocket to poor patients; whereas companions are sometimes engaged to overcome the language barrier or providing support to physically disabled women. Importantly, they admitted they are not equipped with standard (screening) tools to identify such differential needs of patients and also no systematic process to provide supportive care to the patients.

“***Everyone has a different emotional needs*. *They get tensed or cry a lot under stress*, *we call her relatives to stand by them”*. *Lady health visitor*, *Thatta***

### 2 Governance and accountability: Institutional guidelines exist and are combined with effective oversight, regulation, incentives/appraisal and accountability

#### 2.1 Institutional guidelines covering RMC

While non-clinical staff were not aware, all clinical staff reported that they had no guidelines related to RMC at the health facility.

“***We do it [work] according to our understanding*. *Nothing is available about non-medical [interpersonal] care”*. *Gynaecologist*, *Thatta***

However, some job-aids (in the form of posters) were available that explain certain processes of clinical care (like managing PPH, partograph, sterilization of equipment, hand washing etc.). These job-aids were usually given by the non-government organisations (NGOs) as part of short trainings. Notably, these job-aids did not cover anything about RMC–that is, something to reinforce good interpersonal care.

“***There are banners and flip charts mounted everywhere in our hospital given to us by NGOs about Eclampsia*, *corona virus etc*. *But didn’t receive any guidelines from the government [about RMC]”*. *Nurse*, *Thatta***

#### 2.2 Supervision and monitoring

The Obs & Gyne section was visited by in-charge of health facility and external visitors for monitoring purposes especially checking the availability of supplies and medicines, staff attendance, and cleanliness. According to both clinical and non-clinical staff, monitors barely paid attention to the conduct of staff behaviour with patients; and those who did so, they directly took feedback from the patients or their companions. These monitoring visits are made during day time (morning shift), and seldom done during evening and night shifts. It was however reported that immediate actions were taken by the monitors in case a shortcoming is identified during the visit.

“***Supervisors visit our health facility to check the cleanliness and they also check the stuff we receive*. *They fulfil the shortage of medicine”*. *Midwife*, *Thatta***

There was also a suggestion from a clinical staff member that mystery/standardised patients could potentially be a good monitoring approach to assess staff behaviour with patients.

“***You come as a mystery patient and ensure check and balance of staff*. *This would be ideal way to observe staff behaviours with patient*.*” Gynaecologist*, *Thatta***

The administrative leadership of the health facility seemed to be less passionate about improvements in behaviour of maternity staff towards patients especially in a resource constraint health system. According to them the health facility is already short of basic necessities for patients (e.g. medicines) as well as for the working staff (e.g. staff shortage), therefore it is difficult to ensure all staff members keep a good behaviour with patients. They did mentioned though that they occasionally try to reinforce staff to keep good behaviour with the patients; however, unless their staff needs are fulfilled, expecting them to treat all patients with respect and dignity is a big ask. A medical superintendent of health facility said:

“***If we don’t have sufficient resources in health facilities for staff which is distressing for them*. *Then how can we expect them to treat patient with dignity*.*” Medical Superintendent*, *Thatta***

#### 2.3 Roles and responsibilities

Clinical staff members play a specific assigned role in a labour room. In-charge doctor or nurse prepare roster for the staff and patients’ treatment plan; whereas, nurse/midwife provide care accordingly. While, non-clinical staff (especially *Aaya*) ensure necessary equipment are sterilised and in place and ensure cleanliness.

“***I have to look after everything in the labour room*. *Everything should be available for the mother’s care and stuff related to newborn*. *I also look after the patient in the labour room and check all the available staff from time to time”*. *Lady Health Visitor*, *Thatta***

We also observed a lot of un-official task shifting to the non-clinical staff (*Aaya* and cleaner) during our visits to health facilities and was also reported by them. They, support clinical staff during childbirth, and at times, the *Aaya* conduct normal delivery in the absence of clinical staff. And the reason for *Aaya* to willingly assist normal deliveries was the informal payments that they can claim directly from the patients and their companion in addition to their monthly salary.

“***I maintain trolley first and set instruments in the labour room*. *If there are three deliveries at a time*, *then one is handled by doctor*, *another is handled by nurse*, *and I take care of third patient”*. *Aaya*, *Thatta***

No clinical staff member explicitly highlighted: information sharing for informed decision making, ensuring respect and dignity of patient, and provision of psychosocial support as part of their patient care responsibilities. The primary focus was around provision of clinical care.

#### 2.4 Accountability

With regard of patient feedback, no specified mechanism was in place in health facilities. At some places, patients/companions who are aware and empowered or report to senior maternity staff or in-charge of health facility who takes immediate actions without any formal record keeping. Mostly, complaints are lodged in case of serious error or neglect shown by the staff which has caused harm to the patient. Similarly, both clinical and non-clinical staff reported that there is no system to gather women’s feedback about experiences of maternity care. According to them such things only happen in the big private hospitals in the cities.

“***There is no proper [complaint] system here*. *I am the one who listen to all the complaints*. *For example staff didn’t empty urine bag*. *Cleaner is asking for bribe from the patients*. *All these things disturb the patients*. *When they bring the complaints then I talk to the staff*. *If they don’t understand then I scold them”*. *Nurse*, *Thatta***

There was however a general perception that patient complaint could play a role in improving staff behaviour with patients. A lady health visitor in Thatta district said:

“***If we start documenting patient complaint*, *the behaviour of staff will improve because they would know that if they do anything improper*, *there will be consequences*,*” Lady health visitor*, *Thatta***

### 3 Health workforce: A well-performing, competent and motivated health workforce that is responsive to RMC needs given the available resources

#### 3.1 Understanding patient’s rights, respect, and support

Overall, the understanding of participants about patient’s rights, respect and support was low. Most of the clinical staff highlighted that patients’ rights primarily revolve around getting good clinical care such as patients are duly offered timely examinations by the staff (doctors) and given free medicine etc.

“***Whatever government has provided in this institution like medicines*, *beds*, *doctors*, *patients have full right on them*. *It is our duty to provide them these things”*. *Lady health visitor*, *Thatta***

With respect to non-clinical aspect of care, confidentiality/privacy was identified by most respondents as an important right. Very few participants identified good behaviour of staff as a patient right without elaborating as to what are the elements of good behaviour.

“***Patient’s right is to have good behaviour with them*. *We should feel their pain*. *It is our job to explain them that the facilities in this hospital are for you*.*” Nurse*, *Thatta***

For both clinical and non-clinical staff, the concept of respect was primarily linked with speaking politely, demonstrating good behaviour, and feeling others pain. And it was considered as a two-way phenomenon whereby you are respected only when you respect others.

“***It [respect] is necessary for both doctor and patient*. *The person who is coming to our centre*, *we should respect her and she should respect us too”*. *Nurse*, *Thatta***

The connotation of ‘support’ for clinical and non-clinical staff was provision of instrumental/tangible help to patients. Emotional support to patients by showing empathy was identified by very few participants.

“***Patients are physically and mentally disturbed*. *We have deal with her physical condition as well show empathy and good behaviour*. *Mentally fit patients can easily overcome their physical sufferings”*. *Nurse*, *Sujawal***

#### 3.2 Trainings and Staff competency regarding RMC

Almost all of the participants reported that they have not received any training during the past 2 years. Some, however, mentioned about patient counselling training in the past but in the context of family planning.

“***I have received training on…family planning counselling*. *Haven’t received any behaviour related training”*. *Nurse*, *Sujawal***

None of the staff members reported ever receiving any on-job training on interpersonal communication or provision of psychosocial support to patients and their companions.

“***Clinical trainings are sometimes conducted by NGOs*. *There is no such concept of behavioural trainings in government*. *I have never seen such things*. *All health staff have their own way of doing things [dealing with patients]”*. *Nurse*, *Sujawal***

Few clinical staff did mention that they were taught about communication skills and issues around confidentiality and consent during their formal academic courses.

“***Obs and Gyne consultant trainings include communication skills*. *How to counsel the patient*, *how to maintain privacy*, *interpersonal relationship between doctor and patient*, *and Explaining consent nicely”*. *Gynaecologist*, *Thatta***

#### 3.3 Team relationship and coordination

It occurred that the overall relationship between clinical staff was good. In sub-district level health facilities, staff not only support each other professionally.

“***All the doctors are senior*, *we all remain in cooperation such as if one doctor is handling OPD then another doctor would be working in labour”*. *Gynaecologist*, *Sujawal***

However, there was lack of communication and disconnect between clinical and non-clinical staff. A few non-clinical staff (*Aaya* and cleaner) expressed concerns about rude behaviour of senior doctors. They were of the view that everyone plays its role in care provision but they [non-clinical staff] are treated as inferior to clinical staff.

“***People show attitude to me*. *Sometimes even doctors are disgusted by us*. *Why*? *Aren’t we humans*? *Aren’t we like them*? *Why they hate us*? *Here doctors think of themselves as God”*. *Cleaner*, *Sujawal***

The reported coordination among maternity team to deliver good care to patients was reasonable. The junior staff such as non-clinical staff and nurses/midwifes are well-supported by the physicians in case of issues. However, a few staff members raised concerns about delayed or inadequate hand-off from other shifts, and that staff of different shifts do not complete their due tasks which put extra burden on other shifts.

The in-charge of health facility (at sub-districts hospitals) also intervenes to resolve contentious issues around aggravated conflicts with patients and their companions about care provision.

“***Patients are very poor…their attendants become aggressive and this becomes difficult to deal with them*. *In such cases we call MS [in-charge of health facility]*, *so that he can better deal with them”*. *Gynaecologist*, *Sujawal***

#### 3.4 Motivation of maternity team

Majority of the maternity team members reported to be intrinsically motivated towards their job, and were passionate about the kind of work they do–that is, serving people.

“***I like to serve my patients*. *I like to help them*. *Domestic women look after their families and household chores… We are living to serve the humanity*.*” Midwife*, *Thatta***

Some junior and non-clinical staff provided extrinsic reason (mainly monetary benefit) as a main source of doing their job. Another key factor behind motivation which higher their morale is the praise received from patients and their families and appreciation from the seniors.

“***When people go home after delivery*, *they hug me*, *they kiss me Peoples call me a doctor because of my behaviour”*. *Aaya*, *Sujawal***

However, it was reported that appreciation from seniors is seldom happens. And this lack of appreciation from, or at times, degrading behaviour of seniors causes demotivation among staff members. A few clinical staff (mainly physicians) expressed concerns of not being optimally used whereby they are not able to apply their learning in current routine practice.

“***Our administrator has never acknowledged or praised us for our work*. *No matter how good work you do*, *so it hurts a lot*. *He would never praise our work*. *Nurse*, *Sujawal***

### 4 Information system (system that ensure production, analyses, sharing and timely use information to improve health status of patients and performance of health facility)

The information regarding information system was provided by the clinical staff. According to them, the records of patients and stock were maintained manually. There were multiple registers for different phases of care. For example, women in labour gets registered indoor registered; information on women delivered in the health facilities is recorded in Obstetric register etc. Separate registers are maintained for different phases of care.

“***Those who are in labour pain*, *their entry is done in the ‘indoor register’*. *We do the entry of antenatal care*, *high BP patients*. *After normal delivery or C-section*, *their entries are done in Obstetric register”*. *Nurse*, *Thatta***

The record keeping and reporting mechanism resolved around clinical care of patients. The information gathered from patients was solely about their clinical conditions. No provision was found in the registers that captures potential socio-demographic or psychosocial vulnerability and functional disability.

### 5 Health financing (adequate funds to enhance facility environment and service providers capacity to provide RMC)

The funds are kept at the district central office level. There is less flexibility regarding the use of local investment whereby the funds are primarily usually used for salaries, equipment, supplies and maintenance as opposed to capacity building of staff which is often done by non-governmental organisations, or in some cases, senior staff provides on-job training to junior staff.

“***We get limited funds which are spent on infrastructure*, *supplies and medicines*. *The funds for capacity building are kept with the district office and not given to us”*. *Information management officer*, *Thatta***

### 6 Infrastructure and material management: Availability of required material and supplies to provide RMC to the patients

District-level hospitals, by virtue of their design, were better equipped and had greater facilities as compared with sub-district hospitals. However, all health facilities had shortage of curtains, bed linen, adequate seating arrangement for companion, shortage of medicines. A general concern regarding privacy and confidentiality was raised that there is lack of partition in labour room and general architecture of Obs & Gyne section in a hospital.

“***The place where our [OBYGN] section is located is not right*. *There is usually a crowd of males during the day which makes us and pregnant women uncomfortable to come here as this is the only entrance”*. *Midwife*, *Thatta***

## Discussion

**Fig 1:**
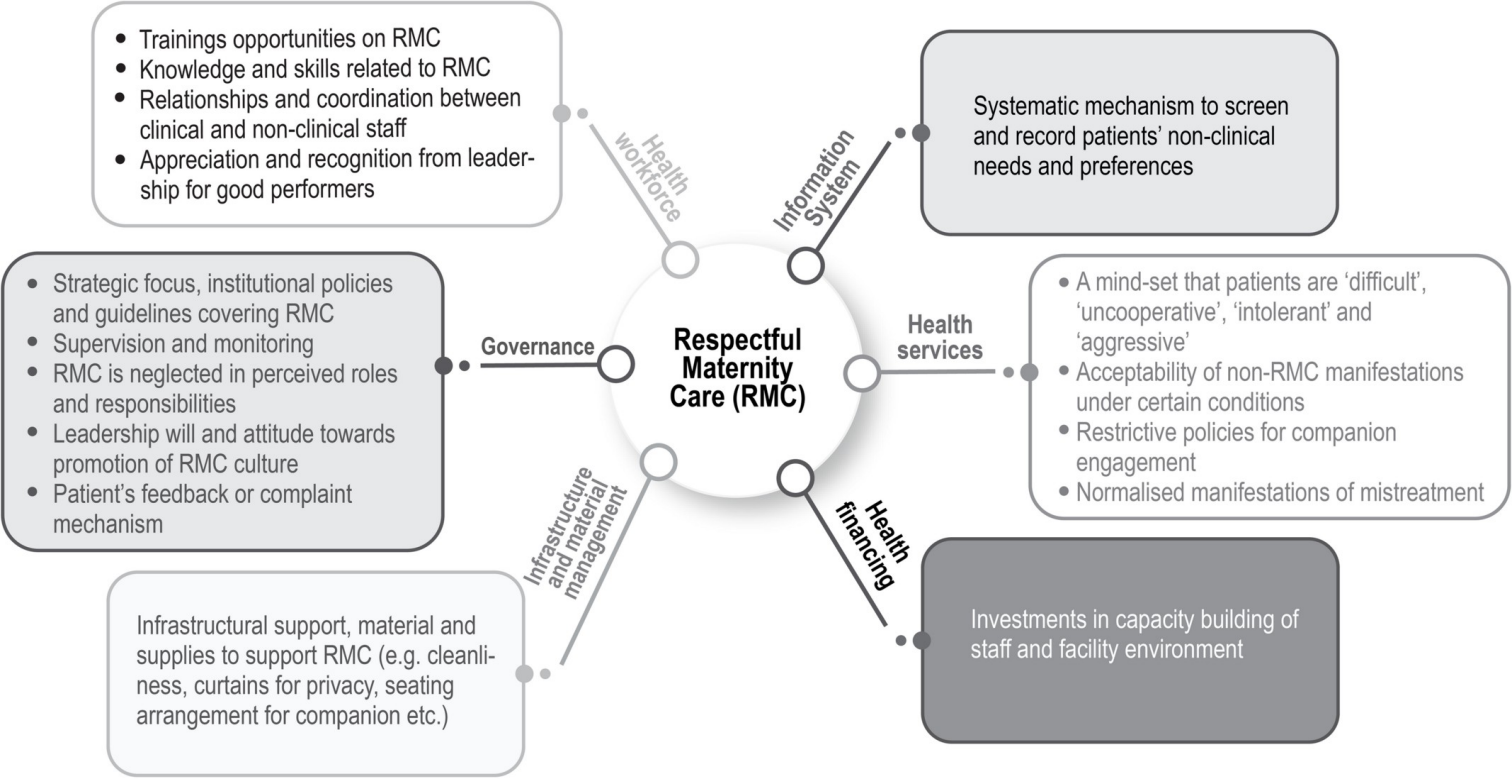
Summary of health systems bottlenecks of respectful maternity care.

Our study investigated the issue of mistreatment during childbirth which revolves around health system responsiveness. We also identified several health system bottlenecks that impedes provision of respectful maternity care in public health facilities (Fig 1). The use to health systems building blocks framework enabled us to specifically identify inputs and processes related issues of the health systems that served as impediments to provision of RMC. Removing these specific bottlenecks across building blocks can make the health system more conducive to embed RMC interventions sustainably.

Broader health system factors that influence RMC is an important but relatively a less researched area. We examined both–broader health system level factors as well the health workers level factors–and trying to relate how the deficiencies in the broader factors may directly or indirectly influence other functional elements of care provision at the individual level. Poor leadership and governance has been linked with mistreatment in earlier studies [12,43]. Our study identified RMC as a blind spot in the overall leadership and governance of the public health facilities. RMC was neither covered in institutional guidelines nor was it emphasised during the monitoring visits conducted by the leadership of health authorities. The monitoring visits focus more on availability of supplies and medicine, staff attendance and cleanliness. Consequently, RMC was found to be completely missing from the perceived role and responsibilities of maternity staff. Without strategic commitment and leadership will, expecting staff to focus on RMC is unrealistic [43]. Therefore, to inculcate a culture of respectful care, it has to be reflected in mandate of good governance, and the leadership needs to effectively engage with staff and set a shared vision [44].

Effective health information system aids evidence-informed decision making that ultimately leads to improvements in quality of care [45]. In terms of RMC, we didn’t find any systematic mechanism in place to enable providers screen for potential psychosocial vulnerabilities among patients. Provision of personalised care would not be possible without proper screening and identifying psychosocial needs and preferences of the pregnant women. Similarly, there was no formalised mechanism in health facilities that empower patients to provide feedback about their experiences of care. In general, patient’s feedback play a pivotal role in quality improvement [46].

In view of the limited resources, there has been lack of monetary investments in capacity building of health care workers on RMC. The focus again has been primarily on enhancing clinical knowledge and skills of healthcare workers. Moreover, consistent with other studies [29], infrastructure and supplies related issues were documented that serve as barriers to provision of RMC. These gaps signify the need for strategic investments in capacity building of staff on RMC and on improvement facility environment to promote RMC.

Our study also identified numerous individual level factors that obstruct RMC. Many of these are a result broader systemic factors. Consistent with other studies [16,17], we observed lack of understanding among both clinical and non-clinical health workers about RMC. Importantly, linking patient’s rights and support merely with tangible items is perhaps not only attributable to lack of training but also lack of RMC-focused supervision and monitoring from higher authorities. Interestingly, the connotation of ‘respect’ was merely confined to ‘talking politely’ to the patient; and a perception that it is two-way (give and take) phenomenon. This perceived notion of respect may be a precursor to mistreatment with pregnant women as many service providers cited that mistreatment is a reaction to the abusive behaviour of patients or their disobedience [16,20]. Coordination and relationship among clinical staff was good. However, there was a clear relationship gap between clinical and non-clinical staff which is a reflection of power dynamics. The sense of inferiority and feeling of helplessness/lack of control particularly among non-clinical staff could instil frustration, and their need to assert power on patient may result in mistreatment [47]. Most staff were reported to be intrinsically motivated and appreciation from women and companions, which is consistent with another study [29]. It is expected that adoption of new behaviours around RMC will result in increased professional self-esteem and sense of motivation for better care–largely due to appreciation from women and their companion [48]. Lack of appreciation from or degrading attitude of seniors / administration was a challenge identified by the maternity staff which indicates the appetite among staff member for appreciation from seniors. Hence, it could be used an opportunity to promote RMC in health facilities by staff training and giving recognition and appreciation of best respectful and supportive carers [49]. Targeted Trainings have shown improvements in understanding of RMC among service providers [50–52].

Un-cooperative and demanding patients and their companions were considered as a major challenges for the staff. In medicine, labelling patient as ‘difficult’ or ‘bad’ or ‘un-cooperative’ has been discussed extensively in the literature [53,54]. In paternalistic approach of care, providers want to be in charge of care, hence acquiescence patients are considered ‘good’ [55]. The non-compliance from patients or companion creates tension between service provider and them. It was basically an interplay of frustration and lack of control–and in such situations providers need to assert power to ensure compliance, resulting in mistreatment [16,20,22,56]. The most serious of abuse (physical or verbal) usually occurs at the time of childbirth where the service providers rationalised that they were compelled to react in a such a manner to save life of a mother and newborn [6,16,20]. The tacitly acceptance and willingness to discuss such actions is indicative of both the normalisation of this behaviour [57,58] as well as lack of process for redress [59].

Persistent unreasonable patients’ demands and repeated explanation were also cited as an underlying causes of mistreatment. While this may be a challenge for service providers in view of low female literature (17%) in our study districts, it also highlights the need for building capacity of service providers on effective communication skills with patients—particularly dealing with difficult situations—which possesses immense significance in healthcare [60,61].

Owing to the psychological distresses pregnant women go through during labour and childbirth, provision of psychological support has been a recommended component of intrapartum care [3]. Our study revealed a clear gap in providers understanding of identification of vulnerable pregnant women (e.g. language barrier, visible disability, anxiety and depression or someone under stress). In view of the growing burden of mental disorder, we suggest formal training of maternity service providers on that front to ensure provision of psychosocial support in a systematic manner after proper screening. Moreover, based on WHO’s recommendation, women’s companion should also be effectively engaged who play an instrumental role in the provision of psychosocial support [62].

Provision of such supportive and dignified maternity care would require a multi-faceted service-delivery intervention package that effectively uses all six components of the health system—from investments in capacity building of maternity teams to creating a conducive facility environment via proper governance and accountability.

### Limitations

Our study suffers from few limitations. First, social desirability bias owing to the topic in focus, as service providers might be reluctant to report malpractices about themselves or colleagues. However, many did acknowledge its occurrence in their setting with different reasoning. Second, our study sample was selected from six secondary-level health from only two districts of southern region, hence findings may not reflect the perspectives of healthcare provider across the country. Also, it cannot be generalised to primary or tertiary-level of healthcare system where the dynamics of routine operations differ considerably. Third, our study excludes perspectives of birthing women about their experiences and expectation of maternity care since the scope was confined to understanding systemic factors. Women’s congruent or contrasting views may have provided deeper understanding of patient-provider interaction issues. However, a strength of the study is that it captured perspective of clinical and non-clinical staff, and health managers across all health facilities. Also, the use of health system framework for RMC was an innovative concept which allowed examining the issue from a uniquely different perspective found uncommon in existing literature.

## Conclusion and recommendations

Our study identified broader health system and individual level bottlenecks according to the six building block of health systems that impedes provision of respectful maternity care. While it is important to reduce manifestations of mistreatment, there is glaring need to promote the culture of psychosocial support for the patients as well as for the staff. There has to be a clear strategic intent on RMC which needs to be communicated by the leadership to the maternity team in the form of standard operating guidelines. For effective implementation, investments should be made to build capacity of service providers on RMC and its interlinked issues especially psychological support. Accountability and encouragement measures such as effective oversight by administration and patient complaint or feedback mechanism should be in place to aid sustained and continuous improvements in RMC at health facilities. Improved management information should serve as a cross-cutting theme which should begin with systematic screening of patients’ needs to support treatment plan, proper documentation of patient feedback about RMC experiences. In a nutshell, promotion of RMC in health facilities requires a holistic health system intervention that addresses bother broader systemic issues as well as individual-level challenges that independently or together hinder provision of RMC within health facilities.

## Supporting information

S1 Text Interview guide for health facility staff (English). (PDF)

S2 Text Interview guide for health facility staff (Urdu). (PDF)

S3 Text Description of routine operations of Obstetrics & Gynaecology section in a secondary-level public health facility. (DOCX)
