# Supplementary material for: Health system bottlenecks hindering provision of supportive and dignified maternity care in public health facilities
Source: PLOS Glob Public Health. 2022 Jul 8;2(7):e0000550. doi: 10.1371/journal.pgph.0000550 (PMC10021678; doi:10.1371/journal.pgph.0000550)
Supplement: S1 Text — (PDF) [file pgph.0000550.s001.pdf]

IN-DEPTH INTERVIEW GUIDE FOR MATERNITY STAFF

S.no	Questions	Responses	Skip
**General Information**			
101	Date of interview	______ / _______ / _____________<br>dd mm yyyy	
102	Name of district	Thatta 1<br>Sujawal 2	
103	Name of TALUKA / Tehsil		
104	Name of Union Council		
105	Name of health facility		
106	Name of health facility / Hospital		
107	Type of study participant	Administrative staff 1<br>Clinical staff 2<br>Non-clinical staff 3	
108	Sex	Male 1<br>Female 2	
109	Age (in years)	Years ☐☐	
110	What is your designation at this health facility?	Medical superintendent 1<br>In-charge- Obs/Gyne Section 2<br>Medical Officer 3<br>Nurse 4<br>Midwife 5<br>Sweeper 6<br>Aaya 7<br>Security guard 8<br>Technician 9<br>Others specify____________________ 10	
111	How long you have been working in this health facility?	Years ☐☐	
112	What is the duration of your total professional experience?	Years ☐☐	
113	Highest level of education completed?		
114	In which shift do you work?	Morning 1<br>Afternoon 2<br>Night 3<br>Others specify________________________	
115	Interview start time	|___|___|:|___|___|	
116	Interview end time	|___|___|:|___|___|	

Interview guide with health facility staff

No.	Questions	Probe
**Q1**	First, I would like to ask you some questions about the health facilities – its infrastructure, staffing, kind services that are offered here, patient volume, available guidelines, and how things are routinely performed and managed here.	
	MAIN THEME: STRUCTURE OF HEALTH FACILITY AND ROUTINE OPERATIONS	
101	What maternal and child health services are offered in this Obs/Gyn department?	*Ask separately about maternal and child services*
102	How many rooms and beds are available? What is the usually average volume of birthing women per month?	**Ask separately about:**<br>• Can you tell me the number of normal deliveries conducted in a month?<br>• Can you tell me the number of C-Section deliveries conducted in a month?<br>• If fixed, what days are fixed for C-Sections?<br>• What days of a month you have high and low volume of births?
103	Can you describe team composition or organogram of Obs/Gyn department?<br>**Draw organogram**	**Ask about:**<br>• What is the number of total staff members?<br>• Who are the supervisors at each level?<br>• How the work shifts are organized?<br>• Who has the decision making authority at each level? And what kinds of decision she can take? Please give example.
104	What kind of clinical and non-clinical trainings are provided to the staff here during the last 2 years?	**Probes:**<br>• Can you tell me the objective of each training?<br>• Who conducted those trainings?<br>• How many times the training was conducted and for whom?<br>• What was the duration of each training?
105	Can you describe how patients' –information is collected and stored?	**Probes:**<br>• Please describe in detail about the type of information that is collected at each stage women go through from admitting to the hospital and all the way to discharge.<br>• Can you tell me about the format/forms that are required to be filled at each stage and who filled these forms?<br>• Where these forms/patient files are stored and retrieved?<br>• If any, what data are entered into the computer?
106	Can you tell me how the patients' information is used for: a) decision making to ensure that appropriate / needed care is provided to the woman; and b) to improve the overall performance to this department?	**Probes:**<br>• Can you give an example how patient data were used to provide appropriate / needed care to the patient?<br>○ Who takes the decision?<br>○ How the decision is taken?<br>• Can you give an example how patient data were used to improve the performance to health facility?

		○ Who takes the decision?<br>○ How the decision is taken?
	**Sub-theme: Quality assurance**	
107	**Now I would like to ask some questions the mechanisms that are in place to ensure quality of services that are provided to the patients. Please note that I'm particularly interested in knowing the care that is provided to women who come here for childbirth.**	
108	What kind of guidelines are available for service provision?	Clinical care; non-clinical (communication, respect, support etc.), record keeping
109	How do you ensure that maternity care services are being provided in accordance with standard operating protocols (SOPs)?	Clinical care:<br>• Are you trained on these SOPs?<br>• Who is responsible for ensuring that everyone adheres to the SOP?<br>• How frequently do they monitor?<br>• How does s/he makes sure compliance (what does s/he look at)<br>• What actions are taken in case of non-compliance?<br>• Who is responsible to address gap in implementation of routine service delivery?<br>• Is there any forum where shortcomings are discussed (e.g. performance review meeting?) If yes, please tell us about it?<br>Non-clinical:<br>• Do the SOPs cover how staff should behave with the patient (e.g. respect, friendliness, effective communication etc?)<br>• How it is ensured in routine practice<br>• What actions are taken in case of non-compliance?
110	What is the role of district health office to ensure quality maternal care in this hospital?	• How the support is provided?<br>• Who is responsible?<br>• How effective it is?
**Q2**	**Theme: Understanding of key concepts**	
	***I would like to gauge your understanding of few terms/concepts that are used in healthcare.* I will show you a picture and will read out few words, and will ask you to explain what you understand by them. Let me remind you that there is nothing right or wrong. We only like to know your opinion.**	
201	Instruction: Please show this picture to the respondent and ask the following questions:<br>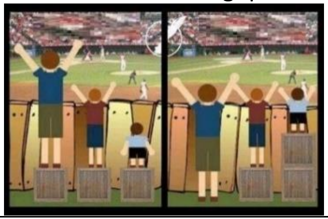	• Can you describe what do you see in this picture?<br>• Can you share some examples from your daily life to explain this picture?<br>• How do you relate this to the care that is provided to women in the labour room and maternity wards?<br>• Do you think women come to this facility for childbirth have varying needs?<br>• How these differential needs are addressed during care provision?<br>• What challenges do you face in addressing these varying needs?

202	How would you define the word 'respect'?	• Can you share some examples from your daily life to explain this word?<br>• How do you relate this to the care that is provided to women in the labour room and maternity wards?<br>• How do you ensure that maternity care is provided in a respectful manner?<br>• What challenges do you face in providing maternity care in respectful manner?
203	What you understanding by the term 'support'?	• Can you share some examples to explain the term in your daily life?<br>• How to you provide support to your family – wife, mother, children?<br>• How do you relate this to the care that is provided to women in this hospital?<br>• What kinds of support is provided to the women during maternity care?
204	What do you understand by the term "medical ethics?"	• Can you share some examples to explain the term<br>• How do you relate this word with the maternity services provided at this health facility? Give some examples<br>• How to you provide support to your family – wife, mother, children?
205	What do you understand by the term "patients' rights"?	• Can you share some examples to explain the term<br>• How do you relate this word with the maternity services provided at this health facility? Give some examples?<br>• How do you ensure that rights of patients are ensured during maternity care?<br>• What challenges do you face in providing right-based maternity care?
206	In your opinion, what are the rights of patients who come to this facility for childbirth?	Share some examples<br>- Right to information (effective communication)<br>- Consented care (informed consent)<br>- Autonomy (respecting women's choices)<br>- Confidentiality / privacy<br>- No verbal abuse (shouting, yelling, threating etc.)<br>- No physical abuse (beating, slapping etc.)<br>- No sexual abuse<br>- No neglect or abandonment (e.g. ignore, delay, refusal)<br>- Non-discriminatory care (e.g. poor care for disabled etc.)<br>- Continuous/supportive care (e.g. companionship)
**Q3**	Theme: Roles and responsibility of staff	
	Now I would like to specifically ask about yourself and your role in this health facility	
301	What is your role / job responsibilities in this hospital?	- Can you describe as per your official job description<br>- What additional work do you do apart from your job description (if any)
302	Describe what your typical day at this health facility looks like. **DAILY ROUTINE – encourage to draw a timeline figure**	**Instructions:** Please ask the respondent to describe each activity in detail from the point they enter and till s/he leaves the health facility.

303	What do you like about your work? Things that keep you motivated?	Why do you like these things?
304	What part of your job do you like the least OR you do not like?	Why do you like it the least?
305	What are the challenges you face in your day-to-day work?<br>First, Can you tell me about the challenges related to your co-workers?	Probes:<br>- Why do you consider these as a challenge?<br>- Why do you think you face those challenges?<br>- How do you deal with or overcome these challenges?<br>- Are there any institutional guidelines/mechanisms to deal with those challenges?<br>- And how effective they are in resolving the issue?<br>- What type of relationship with your colleagues?<br>- In your opinion how can build a better working relationship with your colleagues?<br>-
306	Can you tell me about the challenges related to your supervisors?	- Why do you consider these as a challenge?<br>- Why do you think you face those challenges?<br>- How do you deal with or overcome these challenges?<br>- What are some of the key challenges in implementation of these guidelines?Are there any institutional guidelines/mechanisms to deal with those challenges?<br>- And how effective they are in resolving the issue?<br>- How do you usually official terms with your supervisor or senior?<br>- What do you think how can you improve your working relationship with your supervisor or senior?
307	Can you tell me about the challenges related to your patients?	- Why do you consider these as a challenge?<br>- Why do you think you face those challenges?<br>- How do you deal with or overcome these challenges?<br>- Are there any institutional guidelines/mechanisms to deal with those challenges?<br>- And how effective they are in resolving the issue?
308	Can you tell me about the challenges related to your attendants?	- Why do you consider these as a challenge?<br>- Why do you think you face those challenges?<br>- How do you deal with or overcome these challenges?<br>- Are there any institutional guidelines/mechanisms to deal with those challenges?<br>- And how effective they are in resolving the issue?
309	Can you tell me about the challenges related to the systems like record keeping?	- Why do you consider these as a challenge?<br>- Why do you think you face those challenges?<br>- How do you deal with or overcome these challenges?<br>- Are there any institutional guidelines/mechanisms to deal with those challenges?<br>- And how effective they are in resolving the issue?
4	**Sub-theme: Provider burnout**	

401	Often due to amount or nature of work we become physically and mentally exhausted. As a result of which, we lose interest in our work or felt that the efficiency has reduced. This concept is called "Burnout".<br><br>Did you ever experience this?	
402	When did that happened to you?<br>If your opinion, what the reason for that?	Probe:<br>- Was your supervisor or senior responsible for your burnout? Please explain in detail with example.<br>- Was your co-worker responsible for your burnout? Please explain in detail with example.<br>- Were the patients or their attendants responsible for your burnout? Please explain in detail with example.<br>- Were the stringent rules and regulations of this health facility responsible for your burnout? Please explain in detail with example.
403	How does the burnout affect your work?	Probe:<br>- Your relationship with your senior/supervisor?<br>- Your relationship with your co-workers?<br>Your relationship with your patients or their attendants?
404	How do you cope when you are in the state of burnout?	Please explain in detail with example.
405	What do you suggest how can we mitigate the burnout from maternity staff?	Please explain in detail with example.
**Q5**	Theme: Relationship with co-workers	
	Now I would like to ask about your relationship with your co-workers and supervisors	
501	The challenges you just mentioned, what kind of support do you receive from your co-workers AND supervisors to deal with these challenges?	Ask separately about co-workers and supervisors
502	In general, how would you describe your relationship with your co-workers	Why do you think the relationship is good, ok, or bad
503	In general, how would you describe your relationship with your supervisors	Why do you think the relationship is good, ok, or bad
504	What do you do to make this relationship supportive?	How do you do?<br>Why do you do?
505	What do you expect from co-workers to improve the relationship?	Probe: why do you such expectations
506	What do you expect you're your supervisor to improve the relationship?	Probe: why do you such expectations
	**Sub-theme: suggestions and recommendations**	

407	In your opinion, how can the relationship with co-workers be strengthened or more supportive?	Why do think this strengthen the relationship?
408	In your opinion, how can the relationship with supervisors be strengthened or more supportive?	Why do think this strengthen the relationship?
**Q5**	Theme: Provider interaction with patients	
	Now I would like to ask about patient-provider interaction in this health facility	
	**Sub-theme: Care for women with varying needs**	
501	Can you please describe what happens in a typical case of a delivery?<br>Encourage the participant to develop a flow diagram of the activities?<br>**FLOW DIAGRAM.**	Ask respondents to describe typical journey of a women who comes to deliver the baby at the health facility and then leaves the facility after getting discharged. For example, before delivery in ward, labour room, post-delivery?<br><br>Who deals with women at every stage?<br>How much time do you spend on each phase of care?<br>What kind of care support is provided?<br>How long does a woman spend at every stage?<br>How is it ensure that best care is provided to the woman?
502	Can you describe in details what kind of care is provided to women at each stage?	
503	In your opinion, do all women have similar or different expectations and needs? Can you describe in detail about these differential expectations and needs of women?	Ask separately about physical care and emotional care
504	How do you identify different needs of women?	Personal issues, psychological distress, functional disability, illiteracy, language
505	How do you meet these differential needs and expectations? Can you give examples?	Does someone provide informational support to women?<br>Does someone provide emotional and psychological support?
	**Sub-theme: Challenges in meeting patients' needs**	
506	What are the major challenges you face meeting these varying needs/expectation of women?	What type of difficulties you face providing information?<br>What type of difficulties you face while providing emotional and psychological support?
507	What happens if you fail to meet those expectations and needs?	
	I will now particularly ask about different types of patients that may come to you for childbirth, and would like you to explain how do meet the differential needs to these patients?	
508	How would you deal with a woman who cannot see?	What difficulties do you face dealing with such women?<br>What trainings have you received to deal with such situations?

509	How would you deal with a woman who is physically disabled?	What difficulties do you face dealing with such women?<br>What trainings have you received to deal with such situations?
510	How would you deal with a woman who is scared, anxious and constantly crying?	What difficulties do you face dealing with such women?<br>Have you received any trainings to deal with such situations?
511	How do you deal with woman who cannot understand your language?	What difficulties do you face dealing with such women?<br>Have you received any trainings to deal with such situations?
512	What should be done to deal with these challenges?	Why? How could this implemented?
**Q6**	**Theme: Mistreatment**	
	Now I would like to ask you about patient-provider relationship	
601	Tell me about particular challenging interactions with a women experienced by your colleague. It can be even based on your own experience.	Probes: Do you ever have disagreements with clients?<br>What kind of disagreements?<br>How are they resolved?<br>What hinders provider relationship with clients?<br>Have you been trained on dealing with such situation?
602	Some people say that in public health facilities the behaviour of service providers toward women are is not good? What are your views on that?	Why do you think this way?<br>Have you ever experienced or witnessed tension between care provider and patients?<br>Why was that? How it was dealt?
603	Few people handle the most difficult situation/patient very calmly while ensuring respect and dignity of patient? How do you think they manage it and what is the reason?	
604	Can you suggest changes should be made in this health facility to ensure that varying needs of all women are adequately addressed during intrapartum care?	Instructions: Please ask separately about: capacity building of health facility staff, work ethics, team bonding, institutional policies, and supervision.<br>Probes:<br>- Do you think building capacity of staff could help meeting these needs? Why do you think you? And how this could be done?<br>- Do you think improving work environment for staff could help meeting these needs? Why do you think you? And how this could be done?<br>- Do you think supportive supervision and monitoring could meeting these needs? Why do you think you? And how this could be done?
	**Thank you for your responses. Now I will give you some hypothetical scenarios and will ask you if you were in the given situation how would you deal with the situation.**	
605	A woman who followed a normal pregnancy over nine months has come to the hospital for childbirth. In view	In your current settings, how would you deal with such situation? Why?

	of possible serious complications, the doctor advised the woman to undergo C-section. The woman has refused to undergo the procedure since it was a normal pregnancy and her family members are not in favour of operation. How would you handle the situation?	
606	Due to prolonged labour women has been shouting with severe pain. Despite repeated explanations, the woman is not adhering to the instructions and keep asking for operations. The doctor used harsh tone to deal with her but eventually has slapped the woman.	What are your views about such attitude of doctor? Why do you feel this?<br>Under what circumstances do you think being verbal harsh or handling physically is acceptable?
607	A woman has recently given birth and needs stitching; another women in the final stage of labour needs to be moved to labour room for delivery. However, the anaesthesia is not available. The provider has following choices: a) perform stiches without anaesthesia and free up the bed to other women can come in; b) take a risk and wait for the anaesthesia in this case the other woman may deliver in the ward.	What will be the role of providers in such situation?<br>How would you deal such situation and why?
608	A nurse assisting the doctor has noticed that the doctor is performing post-delivery stitching without anaesthesia and the women is screaming due to pain and complaining not do it. Stopping the doctor may lead to negative consequences for the nurse. What would you if you were that nurse?	How would you deal such situation and why?
609	A woman with normal pregnancy/labour, constantly screaming due to labour pain and asking for pain relief medication. But, the physician remained busy with an emergency in the labour room	How would you deal with such situation and Why?
610	During childbirth, a woman is very restless, not responding to the instructions, and persistently asking for a companion. The person who has accompanied is her husband. Having a companion in labour room is not permissible according to the policy and other women	How would you deal such situation and why?

	are also resisting to due confidentiality issue. How would you deal with the situation?	
611	A 35-year-old anaemic woman after giving birth to a 12th child expressed the desire for tubal ligation, and requested not to inform her family members, as they often physically abuse her, and her husband desires for more children. As per law, the service provider needs to have signed consent from her husband.	How would you deal such situation and why?
612	A 20-year woman giving birth for the first time. Knowing that she is suffering from depression and is very scared of medical procedures, the doctor decides not to share details like what to expect from labour or childbirth and the operative procedure to be used for inducing labour and childbirth. The doctor thinks that it will further disturb her.	What are your views about the decision of the doctor?<br>Under what circumstances do you think such information should not be shared with the women to avoid unrest or panic among women?
**7**	**Sub-theme: Suggestions and recommendation**	
701	What do you suggest which types of initiatives can be taken to improve the relationship between providers and patients? Who and how these initiatives should be taken? If someone asked you what would be your suggestions?	Ask separately about:<br>• Capacity building of health facility staff<br>• Work ethics<br>• Team bonding<br>• Institutional policies<br>• Supervision etc.<br>• Why do you feel this?<br>• How these changes could be incorporated?
702	Anything else that you would like to suggest to improve the maternity care in this hospital?	
