# Supplementary material for: Health system bottlenecks hindering provision of supportive and dignified maternity care in public health facilities
Source: PLOS Glob Public Health. 2022 Jul 8;2(7):e0000550. doi: 10.1371/journal.pgph.0000550 (PMC10021678; doi:10.1371/journal.pgph.0000550)
Supplement: S2 Text — (PDF) [file pgph.0000550.s002.pdf]

تفصیلی انٹرویو گائیڈ برائے میٹرنٹی عملہ

تفصیلی انٹرویو کا سوالنامہ مرکزِ صحت کے عملے کے لیے:

نمبر	سوال	جواب
بنیادی معلومات:		
101	انٹرویو کی تاریخ	|___|___| / |___|___| / |___|___|
102	ضلع کا نام	۱۔ ٹھٹہ ۲۔ سجاول
103	تعلقہ کا نام	
104	یونین کانسل کا نام	
105	مرکزِ صحت کا نام	
106	انٹرویور کا نام	
107	جواب دہندہ کی قسم	1 منیجمنٹ کا عملہ<br>2 طبی عملہ<br>3 معاونتی عملہ
108	جنس	۱۔ مرد ۲۔ عورت
109	عمر (سالوں میں)	|___|___|سال
110	اس مرکزِ صحت پر آپ کا عہدہ کیا ہے؟	۱۔ میڈیکل سپریڈنٹنٹ ۲۔ انچارج—زچہ و بچہ شعبہ<br>۳۔ میڈیکل آفیسر ۴۔ نرس<br>۵۔ مڈوائف ۶۔ ماسی<br>۷۔ چوکیدار/گارڈ<br>دیگر وضاحت کریں__________________
111	آپ کو اس مرکز میں کام کرتے ہوئے کتنا عرصہ ہو گیا ہے؟	سال |___|___|
112	آپ کا پیشہ ورانہ مجموعی تجربہ کتنا ہے؟	سال |___|___|
113	آپ کی تعلیمی قابلیت کتنی ہے؟	
114	آپ کس شفٹ میں کام کرتی/کرتے ہیں؟	۱۔ صبح ۲۔ شام<br>۳۔ رات دیگر وضاحت کریں__________________
115	انٹرویو شروع ہونے کا وقت	|___|___|:|___|___|
116	انٹرویو ختم ہونے کا وقت	|___|___|:|___|___|

نمبر	سوالات	کریدیں
Q1	سب سے پہلے میں آپ سے اس مرکزِ صحت کے بارے میں چند سوالات کرنا چاہوں گی/گا مثلاً شعبہ زچہ و بچہ کا بنیادی ڈھانچہ، عملہ، فراہم کی جانے والے سہولیات کی اقسام، مریضوں کی تعداد، دستیاب رہنما اصول/ ہدایاتی، اور یہ کہ اس مرکزِ صحت میں روزمرہ کس طرح کام انجام دیا جاتا ہے اور انتظامات کس طرح سنبھالے جاتے ہیں۔	
	مرکزی موضوع: مرکزِ صحت کا ڈھانچہ اور معمول کے کام:	
101	اس زچہ و بچہ کے شعبہ میں ماں اور بچے کے لیے کس قسم کی سہولیات فراہم کی جاتی ہیں؟	**ہدایات:** ماں اور بچے کی سہولیات کے بارے میں علیحدہ علیحدہ پوچھیں
102	اس زچہ و بچہ کے شعبہ میں کتنے کمرے اور بستر دستیاب ہیں؟<br>یہاں مہینے میں اوسطاً کتنے بچوں کی پیدائش ہوتی ہے؟	**علیحدہ علیحدہ پوچھیں:**<br>- ایک ماہ میں نارمل پیدائش کتنی ہوتی ہیں؟<br>- ایک ماہ میں آپریشن کے ذریعے کتنی پیدائش ہوتی ہیں؟<br>- کون سے دن آپریشن کے لیے مخصوص ہیں؟<br>- عموماً مہینے کے کون سے دنوں میں زچگی بہت زیادہ یا بہت کم ہوتی ہے؟
103	کیا آپ تفصیلاً بتا ئینگی یہاں کا عملہ کن لوگوں پر مشتمل ہے اور اس کی درجہ بندی کس طرح سے ہے؟<br>**برائے مہربانی آرگینو گرام / یا خاکہ بنا کر بتائیے۔**	**علیحدہ پوچھیں:**<br>- مجموعی طور پر عملے کی تعداد کتنی ہے؟<br>- ہر درجہ پر سپروائیزر کون ہے؟<br>- کام کی شفٹ کس طرح بنائی جاتی ہے؟ مثلاً صبح، شام اور رات کے اوقات کار اور کام کرنے کا عملہ<br>- ہر درجہ پر فیصلہ سازی کا اختیار کس کے پاس ہے؟ اور وہ شخص کس قسم کی فیصلہ سازی کر سکتا ہے؟ کچھ فیصلوں کی مثالیں دیجیے۔
104	گزشتہ ۲ سالوں میں کس قسم کی طبّی اور غیر طبّی ٹریننگ یہاں کے عملے کو دی گئی؟	**کریدیں:**<br>- کیا آپ مجھے ہر ٹریننگ کے مقاصد بتا سکتی ہیں؟<br>- کس کی طرف سے یہ ٹریننگ فراہم کی گی؟<br>- کتنی مرتبہ یہ ٹریننگ منعقد کی گئیں اور کس عملے کے لیے یہ منعقد کی گئیں؟<br>- ان ٹریننگ کا دورانیہ کتنا تھا؟
105	کیا آپ تفصیلاً بیان کر سکتی ہیں کہ مریضوں کی معلومات اکٹھا کرنا اور اس کو محفوظ کرنے کا طریقہ کار کیا ہے؟	**کریدیں:**<br>- برائے مہربانی تفصیلاً بتائیے کہ خواتین کے ہسپتال میں داخلے سے لے کر چھٹی ملنے تک ہر مرحلے پر کس قسم کی معلومات اکٹھا کی جاتی ہیں؟<br>- کیا آپ بتا سکتی ہیں کہ ہر مرحلے پر کس قسم کے فارم/رجسٹر کو پر کرنا ہوتا ہے اور اس کو بھرنے کے ذمہ داری کس کی ہوتی ہے؟<br>- خواتین کے فارم/فائل کہاں محفوظ کیے جاتے ہیں اور ان کو کس طرح ضرورت پڑنے پر کس طرح نکالا جاتا ہے؟

		- خواتین کی معلومات کو کسی کمپیوٹر میں اندراج کیا جاتا ہے؟ اگر ہاں، تو کس قسم کی معلومات کا اندراج کمپیوٹر میں ہوتا ہے؟
106	کیا آپ مجھے بتائینگی کہ خواتین کی معلومات کو فیصلہ سازی کے لیے کس طرح استعمال کیا جاتا ہے؟ ۱۔ فیصلہ سازی مریضوں کی ضروریات کو پورا کرنے کے لیے؛ ۲۔ اس زچہ و بچہ شعبہ کی مجموعی طور پر کارکردگی کو بہتر بنانے کے لیے یا سہولیات کے معیار کو بہتر بنانے کے لیے؟	**کریدیں:**<br>- کیا آپ مثال دے کر بتا سکتی ہیں خواتین کی معلومات کو استعمال کرتے ہوئے اس کو مناسب اور ضرورت کے مطابق دیکھ بھال فراہم کی گئی؟<br>o کون فیصلہ سازی کرتا ہے؟<br>o فیصلہ سازی کا طریقہ کار کیا ہوتا ہے؟<br>- کیا آپ مثال دے کر بتا سکتی ہیں خواتین کی معلومات کو استعمال کرتے ہوئے اس مرکزِ صحت کی کارکردگی کو بہتر بنایا گیا ہو؟<br>o کون اس طرح کی فیصلہ سازی کرتا ہے؟<br>o فیصلہ سازی کا طریقہ کار کیا ہوتا ہے؟
	سہولیات کے معیار کی یقین دہانی	
107	**اب میں آپ سے ان طریقہ کار یا نظام کے بارے میں پوچھنا چاہوں گی/گا جو مریضوں کو فراہم کی جانے والی سہولیات کے معیار کو بہتر بنانے کے لیے بنائے گئے ہیں۔ اس بات کی یقین دہانی کرواتی/کرواتا جائوں کے ہم خاص طور پر ان سہولیات کے بارے میں جاننا چاہیں گے جو زچگی کی خواتین کو فراہم کی جاتی ہیں۔**	
108	سہولیات فراہم کرنے کے لیے کون کون سی رہنما اصول/گائیڈ لائینز موجود ہیں؟	**کریدیں:**<br>طبّی سہولیات (مثلاً سرجری کرنا وغیرہ) اور غیر طبّی سہولیات (بات چیت کا طریقہ کار، مریضوں کا احترام یا معاونت کرنا وغیرہ)، یا مریضوں کا ریکارڈ رکھنے کے حوالے سے پوچھیں۔
109	اس بات کی یقین دہانی کس طرح کی جاتی ہے کہ زچگی کی سہولیات تعین کردہ رہنما اصولوں یا گائیڈ لائینز کے مطابق دی جائے؟	**کریدیں:**<br>**طبّی سہولیات کے حوالے سے:**<br>- کیا آپ کو تعین کردہ رہنما اصولوں کے پر کوئی تربیت دی گئی ہے؟<br>- ہر عملے کو تعین کردہ اصولوں پر عمل درآمد کروانا کس کی ذمہ داری ہے؟<br>- عملہ ان اصولوں پر عمل کرتا ہے یا نہیں کرتا، اس کی نگرانی کس طرح کی جاتی ہے؟<br>- کتنی مرتبہ کی عملے کی نگرانی کی جاتی ہے؟<br>- اگر کوئی تعین کردہ اصولوں پر عمل درآمد نہ کرے تو کس طرح کے اقدامات کئے جاتے ہیں؟<br>- کوئی اس طرح کا فورم ہے جہاں سہولیات کے معیار میں بہتری کے لیے نظر ثانی کی جاتی ہو (مثلاً مہانہ میٹنگ وغیرہ)۔ اس کے بارے میں بتائیے گا۔<br>**غیر طبّی دیکھ بھال:**<br>- کیا کوئی تعین کردہ رہنما اصول ہیں جو یہ بتائیں کہ عملے کو مریضوں کے ساتھ کس طرح کا رویہ رکھنا چاہیے؟ (مثلاً احترام، دوستانہ، موثر بات چیت وغیرہ)

		- اس بات کو کس طرح یقینی بنایا جاتا ہے کہ عملے کا مریضوں کے ساتھ رویہ اچھا ہو۔<br>- اگر کوئی تعین کردہ اصولوں پر عمل درآمد نہ کرے تو کس طرح کے اقدامات کئے جاتے ہیں؟
110	ڈسٹرک ہیلتھ آفیسر اس مرکزِ صحت میں فراہم کی جانے والی زچگی کی سہولیات کو معیاری بنانے کے لیے کیا کردار ہے؟	- ان کی طرف سے کس قسم کی نگرانی یا تعاون کیا جاتا ہے؟<br>- ڈسٹرک کی طرف سے نگرانی یا معاونت کون کرتا ہے؟<br>- ڈسٹرک کی طرف سے نگرانی یا معاونت سہولیات کے معیار کو بہتر بنا کس حد تک موثر ہوتی ہے؟
Q2	موضوع: اہم تصورات کے حوالے سے سمجھ بوجھ	
	**اب میں کچھ اہم تصورات کے حوالے سے آپ کی سمجھ بوجھ جاننا چاہوں گا جو صحت کے حوالے سے جن کا استعمال ہوتا ہے۔ میں آپ کو ایک تصویر دکھاؤں گی/گا اور کچھ الفاظ آپ کے سامنے پڑھوں گی، آپ سے کہوں گی/گا کہ آپ اس کو اپنی سمجھ کے مطابق بیان کریں۔ میں آپ کو دوبارہ یاددہانی کروادوں کہ اس میں کچھ صحیح یا غلط کی بات نہیں ہو رہی ہے۔ ہم صرف آپ کی رائے جاننا چاہتے ہیں۔**	
201	ہدایت: برائے مہربانی اس تصویر کو جواب دہندہ کو دکھائیں اور دیے گئے سوالات پوچھیں:<br>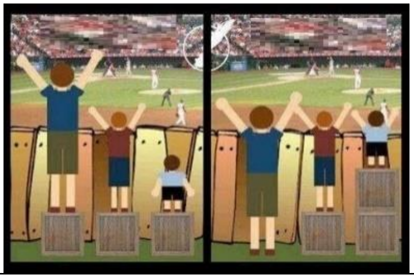	- کیا آپ بیان کر سکتے ہیں آپ کو اس تصویر میں کیا دکھایا گیا ہے؟<br>- کیا آپ اپنی روزمرہ کی زندگی سے کوئی مثال دے کر اس تصویر کو بیان کر سکتی ہیں؟<br>- آپ اس تصویر کو اس مرکز صحت میں فراہم کی جانے والی زچہ و بچہ کی سہولیات سے منسلق کر سکتی ہیں؟<br>- آپ کی رائے میں اس مرکز صحت پر زچگی کے لیے آنے والی خواتین کی ضروریات مختلف ہوتی ہیں؟<br>- آپ ہر خاتون کی مختلف ضرورت کو کس طرح پورا کرتی ہیں؟<br>- آپ کو ان کی مختلف ضروریات کو پورا کرنے میں کن دشواریوں کو سامنا ہوتا ہے؟
202	آپ کے مطابق لفظ 'احترام' سے کیا مراد ہے؟	- کیا آپ اپنی روزمرہ کی زندگی سے کوئی مثال دے کر اس لفظ کو بیان کر سکتی ہیں؟<br>- آپ اس لفظ کو اس مرکز صحت میں فراہم کی جانے والی زچہ و بچہ کی سہولیات سے منسلق کر سکتی ہیں؟<br>- آپ اس بات کی یقین دہانی کیسے کرتی ہیں کہ زچگی کی سہولیات احترام کے ساتھ فراہم کی جائیں۔<br>- اس بات کی یقین دہانی کہ زچگی کی خواتین کے ساتھ عزت و احترام کا رویہ رکھا جائے، آپ کو کن دشواریوں کو سامنا ہوتا ہے؟
203	آپ کے مطابق لفظ 'تعاون'/معاونت/مدد سے کیا مراد ہے؟	- کیا آپ اپنی روزمرہ کی زندگی سے کوئی مثال دے کر اس لفظ کو بیان کر سکتی ہیں؟<br>- آپ اپنی فیملی (بیوی/شوہر، بچے، ماں وغیرہ) کی کس طرح مدد/معاونت کرتے ہیں؟<br>- آپ اس لفظ کو اس مرکز صحت میں فراہم کی جانے والی زچہ و بچہ کی سہولیات سے منسلق کر سکتی ہیں؟<br>- زچگی کی خواتین کے ساتھ کس قسم کی معاونت/مدد کی جاتی ہے؟ کیا آپ کچھ مثالیں دی سکتی ہیں؟
204	آپ کے مطابق لفظ 'صحت کی دیکھ بھال میں اخلاقیات" سے کیا مراد ہے؟	- کیا آپ کچھ مثالوں کی مدد سے اس کو بیان کر سکتی ہیں؟<br>- آپ اس لفظ کو اس مرکز صحت میں فراہم کی جانے والی زچہ و بچہ کی سہولیات سے منسلق کر سکتی ہیں؟ کیا آپ کچھ مثالیں دی سکتی ہیں؟

205	آپ کے مطابق لفظ ‘مریضوں کے حقوق" سے کیا مراد ہے؟	- کیا آپ کچھ مثالوں کی مدد سے اس کو بیان کر سکتی ہیں؟<br>- آپ اس لفظ کو اس مرکز صحت میں فراہم کی جانے والی زچہ و بچہ کی سہولیات سے منسلق کر سکتی ہیں؟ کیا آپ کچھ مثالیں دی سکتی ہیں؟<br>- آپ اس بات کی یقین دہانی کیسے کرتی ہیں کہ زچگی کی سہولیات فراہم کرنے کے دوران مریضوں کے حقوق کا خیال رکھا جائے؟<br>- زچگی کی سہولیات فراہم کرنے کے دوران مریضوں کے حقوق پورے کرنے کے لیے آپ کو کن دشواریوں کو سامنا ہوتا ہے؟
206	آپ کے خیال میں خواتین صحت کی سہولیات کے حوالے سے کون کون سے حقوق میں جو اس مرکز صحت پر زچگی کروانے آتی ہیں؟	کریدنے کے لیے کچھ حقوق مندرجہ ذیل ہیں:<br>- معلومات کا حق (موثر بات چیت)<br>- رضامندی (مریض سے جسمانی معائنہ سے پہلے رضامندی لینا)<br>- خود مختاری (مریض کی مرضی کا احترام کرنا)<br>- رازداری<br>- بدکلامی نہ کرنا (مریض پر چیخنا، چلانہ، دھمکانہ وغیرہ)<br>- جسمانی تشدد نہ کرنا (مریض کو چانٹا مارنا وغیرہ)<br>- جنسی تشدد نہ کرنا<br>- تاخیر یا نظرانداز یا تنہا نہ چھوڑنا<br>- غیر امتیازی سلوک<br>- معاونت/مدد
Q3	موضوع: عملے کا کردار اور ذمہ داریاں	
	**اب میں آپ سے اس مرکزِ صحت میں آپ کا کردار اور ذمہ داریوں کے بارے میں پوچھنا چاہوں گی۔**	
301	آپ کی اس مرکزِ صحت میں کیا ذمہ داریاں ہیں؟	- سرکاری طور پر آپ کی کیا ذمہ داریاں ہیں۔<br>- اپنی سرکاری ذمہ داریوں کے علاوہ کیا آپ کوئی اضافی کام کرتی ہیں؟
302	عموماً آپ کا دن اس صحت کے مرکز میں کیسے گزرتا ہے؟ کیا آپ تفصیلاً بیان کریں گی؟<br>جوابدہندہ کی حوصلہ افزائی کریں کے وہ وقت کے حساب سے ایک خاکہ بنائے۔<br>TIMELINE	**ہدایت: برائے مہربانی جواب دہندہ سے کہیں کو وہ اپنے ہر کام کے بارے میں بتائیں—اس مرکز میں داخل ہونے سے لے کر گھر واپس جاتے تک۔**
303	آپ کا اپنے کام کے بارے میں کیا اچھا لگتا ہے؟	آپ کو اپنے کام میں یہ چیزیں کیوں پسند ہیں۔

	وہ کون سے چیزیں ہیں جو آپ کے لیے حوصلہ افزاء ہیں؟	
304	آپ کو اپنے کام کے حوالے سے کون سی چیز سب سے کم پسند ہے یا اچھی نہیں لگتی؟	آپ کو اپنے کام میں یہ چیزیں کیوں پسند نہیں ہیں۔
305	آپ کو اپنے روز مرہ کی کام میں کن مشکلات کا سامنا کرنا پڑتا ہے؟<br>سب سے پہلے، کیا آپ مجھے یہ بتا سکتی ہیں کہ آپ کو اپنے ساتھیوں کے حوالے سے کن مشکلات کا سامنا کرنا پڑتا ہے؟	**کریدیں:**<br>- آپ کو کیوں لگتا ہے کہ یہ آپ کے لیے مشکلات ہے؟<br>- آپ کے خیال میں آپ کو ان مشکلات کا سامنا کیوں کرنا پڑتا ہے؟<br>- آپ ان مشکلات سے کس طرح نمٹتی ہیں؟<br>- اس مرکزِ صحت پر اس قسم کی مشکلات سے نمٹنے کے لیے کوئی واضح طریقہ کار یا رہنما اصول ہیں؟<br>- یہ طریقہ کار یا رہنما اصول ان مشکلات کو حل کرنے میں کس حد تک موثر ہیں۔<br>- عموماً آپ کے اپنے ساتھی ورکر کے ساتھ کیسے تعلقات ہیں۔<br>- آپ کے خیال میں اپنے ساتھی ورکر کے ساتھ تعلقات کو کس طرح بہتر بنایا جا سکتا ہے؟
306	کیا آپ مجھے یہ بتا سکتی ہیں کہ آپ کو اپنے سپر وائزر یا سینئر کے حوالے سے کن مشکلات کا سامنا کرنا پڑتا ہے؟	**کریدیں:**<br>- آپ کو کیوں لگتا ہے کہ یہ آپ کے لیے مشکلات ہے؟<br>- آپ کے خیال میں آپ کو ان مشکلات کا سامنا کیوں کرنا پڑتا ہے؟<br>- آپ ان مشکلات سے کس طرح نمٹتی ہیں؟<br>- اس مرکزِ صحت پر اس قسم کی مشکلات سے نمٹنے کے لیے کوئی واضح طریقہ کار یا رہنما اصول ہیں؟<br>- یہ طریقہ کار یا رہنما اصول ان مشکلات کو حل کرنے میں کس حد تک موثر ہیں۔<br>- عموماً آپ کے اپنے سپر وائزر یا سینئر کے ساتھ کیسے تعلقات ہیں۔<br>- آپ کے خیال میں اپنے سپر وائزر یا سینئر کے ساتھ تعلقات کو کس طرح بہتر بنایا جا سکتا ہے؟
307	کیا آپ مجھے یہ بتا سکتی ہیں کہ آپ کو مریضوں کے حوالے سے کن مشکلات کا سامنا کرنا پڑتا ہے؟	**کریدیں:**<br>- آپ کو کیوں لگتا ہے کہ یہ آپ کے لیے مشکلات ہے؟<br>- آپ کے خیال میں آپ کو ان مشکلات کا سامنا کیوں کرنا پڑتا ہے؟<br>- آپ ان مشکلات سے کس طرح نمٹتی ہیں؟<br>- اس مرکزِ صحت پر اس قسم کی مشکلات سے نمٹنے کے لیے کوئی واضح طریقہ کار یا رہنما اصول ہیں؟<br>- یہ طریقہ کار یا رہنما اصول ان مشکلات کو حل کرنے میں کس حد تک موثر ہیں۔

308	کیا آپ مجھے یہ بتا سکتی ہیں کہ آپ کو مریضوں کے ساتھ آنے والے لوگوں کے حوالے سے کن مشکلات کا سامنا کرنا پڑتا ہے؟	کریدیں:<br>- آپ کو کیوں لگتا ہے کہ یہ آپ کے لیے مشکلات ہے؟<br>- آپ کے خیال میں آپ کو ان مشکلات کا سامنا کیوں کرنا پڑتا ہے؟<br>- آپ ان مشکلات سے کس طرح نمٹتی ہیں؟<br>- اس مرکزِ صحت پر اس قسم کی مشکلات سے نمٹنے کے لیے کوئی واضح طریقہ کار یا رہنما اصول ہیں؟<br>- یہ طریقہ کار یا رہنما اصول ان مشکلات کو حل کرنے میں کس حد تک موثر ہیں۔
309	کیا آپ مجھے یہ بتا سکتی ہیں کہ آپ کو اس مرکز صحت کے قائدے اور قوانین (مثلاً ریکارڈ رکھنا، آفس کے اوقات وغیرہ) کے حوالے سے کن مشکلات کا سامنا کرنا پڑتا ہے؟	کریدیں:<br>- آپ کو کیوں لگتا ہے کہ یہ آپ کے لیے مشکلات ہے؟<br>- آپ کے خیال میں آپ کو ان مشکلات کا سامنا کیوں کرنا پڑتا ہے؟<br>- آپ ان مشکلات سے کس طرح نمٹتی ہیں؟<br>- اس مرکزِ صحت پر اس قسم کی مشکلات سے نمٹنے کے لیے کوئی واضح طریقہ کار یا رہنما اصول ہیں؟<br>- یہ طریقہ کار یا رہنما اصول ان مشکلات کو حل کرنے میں کس حد تک موثر ہیں۔
4	موضوع: پرووائیڈر کا برن آؤٹ ہونا	
401	عموماً کام کی زیادتی یا کام کی نوعیت، کی وجہ سے ہم جسمانی یا ذہنی طور پر نڈھال ہو جاتے ہیں۔ جسکی وجہ سے ہماری کام میں دلچسپی کم ہو جاتی ہے اور ہمیں کبھی لگتا ہے کہ ہمارے کام کرنے کی صلاحیت میں کمی آئی ہے۔<br>اس تصور کو ہم انگریزی میں Burnout کہتے ہیں۔<br><br>کیا آپ کے ساتھ کبھی ایسا ہوا ہے؟	
402	ایسا آپ کے ساتھ کب ہوا؟<br>آپ کے خیال میں اس کی وجہ کیا تھی؟	کریدیں:<br>کیا سپروائزر یا سینئر آپ کے لیے Burnout کی وجہ بنی۔<br>کیا آپ کے ساتھی آپ کے لیے Burnout کی وجہ بنی۔<br>کیا مریض یا ان کے ساتھ موجود لوگ آپ کے لیے Burnout کی وجہ بنے۔<br>کیا ادارے کے قانون یا قائدے آپ کے لیے Burnout کی وجہ بنے۔

403	Burnoutآپ کے کام پر کس طرح اثرانداز ہوا۔	کریدیں:<br>آپ اپنے سپروائزر یا سینئر کے ساتھ تعلق پر Burnout کیسے اثرانداز ہوا۔<br>آپ اپنے ساتھی ورکر کے ساتھ تعلق پر Burnout کیسے اثرانداز ہوا۔<br>مریض یا ان کے ساتھ موجود لوگ کے ساتھ تعلق پر Burnout کیسے اثرانداز ہوا۔
404	جب Burnoutہوتا ہے تو آپ اس سے کس طرح نمٹتی ہیں۔	کیا آپ مثال دی کر بتا سکتی ہیں؟
405	زچہ و بچہ کے عملے کوBurnoutسے بچانے کے لیے آپ کیا تجاویز دیں گی۔	آپ کو ایسا کیوں لگتا ہے؟
Q5	موضوع: پرووائیڈر کے مریضوں کے ساتھ تعلقات	
	**اب میں آپ سے پرووائیڈر اور مریضوں کے درمیان تعلقات**	
	**Sub-theme: Care for women with varying needs**	
501	کیا آپ تفصیلاً بتا ئینگی کی عام طور پر بچے کی پیدائش کن مراحلے سے گزرتے ہوئے ہوتی ہے؟<br>جوابدہندہ کی حوصلہ افزائی کریں کہ وہ ہر مرحلے کو کاغذ پر نقش کر کے بتائے؟<br>FLOW DIAGRAM	جواب دہندہ سے کہیں کہ وہ خواتین کا سفر تفصیلی بیان کریں۔اس کے مرکز میں آنے سے لے کر ہسپتال سے چھٹی ملنے تک وہ کن مراحل سے گزرتی ہے—مثلاً زچگی کے پہلے، زچگی کے دوران، اور زچگی کی بعد۔<br>- ہر مرحلے پر کس قسم کی دیکھ بھال فراہم کی جاتی ہے؟<br>- ہر مرحلے پر خاتون کو کتنا وقت لگتا ہے؟<br>- اس بات کی یقین دہانی کہ خاتون کو بہترین دیکھ بھال ملے؟
502	ہر مرحلے پر خواتین کو کس قسم کی مدد فراہم کی جاتی ہے؟	
503	آپ کے خیال میں کیا تمام خواتین کی ایک جیسی یا مختلف توقعات یا ضروریات ہوتی ہیں؟<br>کیا آپ بتا ئینگی کی ان کی کس قسم کی مختلف توقعات اور ضروریات ہوتی ہیں؟	کریدیں:<br>جسمانی طور پر اضافی ضروریات اور ذہنی طور پر اضافی ضروریات کے بارے میں علیحدہ علیحدہ پوچھیں۔
504	آپ خواتین کی مختلف ضروریات کی کس طرح نشاندہی کرتے ہیں اور اس کو کس طرح پورا کرتے ہیں؟	- ذہنی دباؤ کا شکار خواتین، معذوری، ناخواندگی، زبان سے واقفیت کا نہ ہونا
505	خواتین کی مختلف توقعات یا ضروریات کو کس طرح پورا کیا جاتا ہے؟ کیا آپ مثال دی کر بتا سکتی ہیں؟	کیا ان کو کوئی معلومات فراہم کی جاتی ہے<br>ان کو جذباتی یا سماجی (نفسیاتی) مدد دی جاتی ہے

	Sub-theme: Challenges in meeting patients' needs	
506	آپ کو خواتین کی مختلف توقعات یاضروریات کو پورا کرنے میں کن مشکلات کا سامنا کرناپڑتا ہے؟	ان کو معلومات فراہم کرنے میں کیا مشکلات ہوتی ہیں<br>ان کو جذباتی یا سماجی (نفسیاتی) مدد دینے میں کیا مشکلات ہوتی ہیں
507	اگر آپ خواتین کی مختلف توقعات یاضروریات کو پورا نہ کر سکیں تو کیا ہوتا ہے؟	
	**اب میں آپ کو کچھ مثالیں دوں گی/گا اور پوچھوں گا کہ آپ اس قسم کی مریضوں کی توقعات پر کیسے پورا اترتی ہیں یا ان کی اضافی ضروریات کو پورا کرتی ہیں؟**	
508	آپ ان خواتین کی کس طرح دیکھ بھال کرتی ہیں جن کی بینائی نہیں ہوتی؟	اس طرح کی خواتین کی دیکھ بھال فراہم کرنے میں کن مشکلات کا سامنا کرناپڑتا ہے؟<br>اس طرح کی خواتین کی دیکھ بھال فراہم کرنے کے لیے کوئی تربیت آپ کو دی گئی ہے؟
509	آپ ان خواتین کی کس طرح دیکھ بھال کرتی ہیں جن کو جسمانی معذوری ہے؟	اس طرح کی خواتین کی دیکھ بھال فراہم کرنے میں کن مشکلات کا سامنا کرناپڑتا ہے؟<br>اس طرح کی خواتین کی دیکھ بھال فراہم کرنے کے لیے کوئی تربیت آپ کو دی گئی ہے؟
510	آپ ان خواتین کی کس طرح دیکھ بھال کریں گی جو بہت ہی ڈری ہوئی ہوں، یا بے چین یا گھبرائی ہوئی ہوں یا مستقل رو رہی ہوں؟	اس طرح کی خواتین کی دیکھ بھال فراہم کرنے میں کن مشکلات کا سامنا کرناپڑتا ہے؟<br>اس طرح کی خواتین کی دیکھ بھال فراہم کرنے کے لیے کوئی تربیت آپ کو دی گئی ہے؟
511	آپ ان خواتین کی کس طرح دیکھ بھال کریں گی جو آپ کی زبان نہ جانتی ہو؟	اس طرح کی خواتین کی دیکھ بھال فراہم کرنے میں کن مشکلات کا سامنا کرناپڑتا ہے؟<br>اس طرح کی خواتین کی دیکھ بھال فراہم کرنے کے لیے کوئی تربیت آپ کو دی گئی ہے؟
512	ان مشکلات سے نمٹنے کے لیے کس قسم کی اقدامات کرنے چاہیے؟	آپ کو کیوں لگتا ہے کہ یہ اقدامات موثر ہوں گے؟<br>ان اقدامات کو اس مرکزِ صحت میں کس طرح لاگو کیا جاسکتا ہے؟
Q6	بدسلوکی	
	اب میں آپ کے پروائیڈر اور مریضوں کے تعلقات کے بارے میں جاننا چاہوں گی/گا۔	
601	مجھے اپنا یا اپنے کسی ساتھی کا کوئی ایسا تجربہ بتائیں جس میں حاملہ خواتین کو زچگی کی سہولیات فراہم کرنے میں بہت مشکلات کا سامنا کرناپڑا ہو۔	کیا آپ کے کبھی مریضوں کے ساتھ اختلافات ہوئے ہیں؟<br>کس قسم کے اختلافات؟<br>ان کو کس طرح حل کیا جاتا ہے؟<br>مریض اور دیکھ بھال فراہم کرنے والوں کے درمیان اچھے تعلق بنانے میں کونسی رکاوٹیں ہوتی ہیں؟<br>کیا آپ کو اس قسم کی مشکل صورت حال سے نمٹنے کے لیے کوئی تربیت ملی ہے؟

602	کہاجاتاہے کہ سرکاری ہسپتالوں میں عملے کارویہ مریضوں کے ساتھ اچھا نہیں ہوتا۔اس بارے میں آپ کی کیارائے ہے؟	آپ کوایسا کیوں لگتاہے؟<br>کیاآپ نے مریض اورپروائیڈر کے درمیان تناؤدیکھاہے؟<br>اس کی کیاوجہ تھی اوراس کو کس طرح حل کیاگیا؟
603	کچھ لوگ ایسے ہوتے ہیں جو کسی بھی مشکل صورت حال یامریض کو بہت ہی اطمینان کے ساتھ حل کر لیتے ہیں،اس بات کاخیال رکھتے ہوئے مریض کی ساتھ کسی قسم کی بدتمیزی نہ ہو؟آپ کے خیال میں وہ لوگ کس طرح یہ باآسانی کرلیتے ہیں اوراس کی کیاوجوہات ہوسکتی ہیں؟	
604	کیاآپ بتاسکتی ہیں کہ اس مرکزِصحت میں کس قسم کی تبدیلیوں کی ضرورت ہے تاکہ زچگی کے لیے آنے والی خواتین کی مختلف توقعات اورضروریات پرپورااتراجاسکے؟	ہدایت:برائے مہربانی علیحدہ علیحدہ بتائیں:عملے کی تربیت؛کام کرنے کاطریقہ کار؛ٹیم کی درمیان بہتر ہم آہنگی؛ادارے کی پالیسی اور بہتر نگرانی۔ کریدیں:<br>- آپ کے خیال میں عملے کو کس قسم کی تربیت دینے کی ضرورت ہے؟آپ کوایسا کیوں لگتاہے؟یہ کس طرح ممکن ہوسکتاہے؟<br>- آپ کے خیال میں مریضوں کی توقعات پرپورااترنے کے لیے کام کاماحول بہتری بنانے کی ضرورت ہے؟آپ کوایسا کیوں لگتاہے؟یہ کس طرح ممکن ہوسکتا ہے؟<br>- آپ کے خیال میں مریضوں کی توقعات پرپورااترنے کے لیے سہولیات فراہمی کی عمل کی نگرانی کی ضرورت ہے؟آپ کوایسا کیوں لگتاہے؟یہ کس طرح ممکن ہوسکتاہے؟
	**آپ کے جوابات کاشکریہ۔اب میں آپ کوکچھ فرضی خاکے بتاؤں گی/گااور جانناچاہوں گاکہ آپ اس صورت حال کو کس طرح حل کرتے۔**	
605	ایک خاتون ہسپتال میں آتی ہے جس کا۹ماہ تک نارمل حمل رہاہے۔اس کی حالت دیکھنے کے بعد ڈاکٹر کو کچھ پیچیدگی نظرآتی ہے اوروہ اس کوآپریشن کروانے کی تجویزدیتاہے۔مگر خاتون منع کردیتی ہے کیوں کہ اس کوپتاہے کہ حمل نارمل ہے اوراس کے گھروالے بھی آپریشن سے منع کردیتے ہیں۔اس صورت میں پروائیڈر کو کیاکرناچاہیے؟	آپ اس صورت حال میں کیاکریں گی اور کیوں؟
606	طویل لیبر کی وجہ سے ایک خاتون درد سے چلارہی ہے۔اس کو کئی بار سمجھایاگیامگروہ نہیں مانتی۔ڈاکٹر اس پر چلائی مگر پھر بھی اس نے بات نہیں مانی۔آخرکار ڈاکٹر نے اس کوایک تھپڑمارا۔	ڈاکٹر کے رویہ کے حوالے سے آپ کی رائے کیاہے؟آپ کوایسا کیوں لگتاہے۔<br>آپ کے خیال میں کن صورت حال میں خاتون سے سختی سے پیش آنایاجسمانی طورپراس کو سمبھالناقبل قبول ہے؟اور کیوں؟
607	ایک خاتون کی ابھی زچگی ہوئی ہے اوراس کوٹانکے لگنے ہیں۔ایک اورخاتون کے بچے کی پیدائش ہونے کے قریب ہے،جس کوفوراًلیبر کے کمرے میں جگہ چاہیے۔اورجب کے سن کرنے کی دواموجود نہیں	اس صورت میں پروائیڈر کو کیاکرناچاہیے؟<br>آپ اس صورت حال میں کیاکریں گی اور کیوں؟

	ہے جو ٹانکوں سی پہلے لگائی جاتی ہے۔ پرووائیڈر کے پاس دو آپشن ہیں: a) بناء سن کیے ٹانکے لگا کر بستر دوسری خاتون کو دے دے؛ b) خطرہ مول لے سن کرنے والی دوائی آنے کا انتظار کرے جس صورت میں دوسری خاتون کی زچگی بھی ہو سکتی ہے۔	
608	ایک نرس، ڈاکٹر کے ساتھ ڈیوٹی پر ہے۔ وہ یہ دیکھتی ہے کہ ڈاکٹر نہیں بچے کی پیدائش کے بعد بغیر سن کیے خاتون کو ٹانکے لگا رہی ہے اور خاتون درد سے چلا رہی ہے۔ نرس کے پاس دو آپشن ہیں: a) ڈاکٹر کو بغیر دوا ٹانکے لگانے سے منع کرے جو اس کے لیے برا ہو سکتا ہے یا وہ خاموش رہے۔<br>اس صورت میں پرووائیڈر کو کیا کرنا چاہیے؟	آپ اس صورت حال میں کیا کریں گی اور کیوں؟
609	ایک نارمل حمل کی خاتون لیبر میں مسلسل چلا رہی ہے اور درد کم کرنے لیے دوا مانگ رہی ہے۔ مگر ڈاکٹر لیبر کے کمرے میں ایک ہنگامی کیس کو دیکھ رہا ہے۔ اس صورت میں پرووائیڈر کو کیا کرنا چاہیے؟	آپ اس صورت حال میں کیا کریں گی اور کیوں؟
610	زچگی کے دوران ایک خاتون بہت بے چین ہے اور دی گئی ہدایات پر بالکل بھی عمل نہیں کر رہی اور مسلسل کہہ رہی ہے کہ اس کے ساتھی کو اندر بلایا جائے۔ جو شخص اس کے ساتھ آیا ہے وہ اس کا شوہر ہے۔ قائدے کے مطابق ساتھی لیبر کے کمرے میں نہیں جا سکتا اور لیبر کے کمرے میں موجود دیگر خواتین منع کر رہی ہیں کیونکہ یہ ان کے لیے رازداری کا مسئلہ ہے۔ اس صورت میں پرووائیڈر کو کیا کرنا چاہیے؟	آپ اس صورت حال میں کیا کریں گی اور کیوں؟
611	ایک ۳۵ سالہ خاتون جس میں خون کی کمی ہے اپنے ۱۲ بچے کی پیدائش کے بعد ڈاکٹر سے کہتی ہے کہ اس کی نل بندی کر دی جائے۔ وہ یہ بھی بتاتی ہے کہ اس کے گھر والے اس پر تشدد کرتے ہیں اور ان کو مزید بچوں کی خواہش ہے۔ پالیسی کے مطابق پرووائیڈر کو شوہر سے اظہارِ رضامندی پر دستخط لینا لازمی ہے۔ اس کے بغیر وہ نل بندی نہیں کر سکتی۔	آپ اس صورت حال میں کیا کریں گی اور کیوں؟
612	ایک ۲۰ سال کی خاتون پہلی مرتبہ زچگی کروا رہی ہیں اور وہ آپریشن یا سرجری سے بہت ڈری ہوئی ہے۔ ڈاکٹر یہ جانتے ہوئے کہ خاتون کو ڈپریشن ہے، یہ فیصلہ کرتا ہے کہ اس کو زچگی کے مرحلے اور اس کے طریقہ کار کے بارے میں نہ بتایا جائے تاکہ اس کو مزید پریشانی نہ ہو۔	ڈاکٹر کے اس عمل کے حوالے سے آپ کی رائے کیا ہے؟ آپ کو ایسا کیوں لگتا ہے؟<br>آپ کے خیال میں کن صورت حال میں یہ بہتر ہے کہ خاتون کے ساتھ معلومات فراہم نہ کرنا قابل قبول ہے؟ اور کیوں؟

8	تجاویز:	
801	اس مرکزِ صحت میں آپ کے خیال میں مریض اور عملے کے درمیان تعلق کو بہتر بنانے کے لیے کس قسم کے اقدامات کرنے کی ضرورت ہے؟ کس کی طرف سے ایسے اقدامات کیے جانے چاہیے اور کیسے؟ اگر آپ سے پوچھا جائے تو آپ کیا کریں گے؟	**علیحدہ پوچھیں:**<br>- عملہ کی صلاحیتوں کو بڑھانے کے لیے<br>- کام کے اخلاقیات<br>- ٹیم کے درمیان بہتر ربط<br>- مرکز صحت کی پالیسی<br>- سپروائز کرنا<br>- آپ کو ایسا کیوں لگتا ہے؟<br>- یہ تبدیلیاں اس مرکز صحت میں کس طرح کی جاسکتی ہیں؟
802	آپ کے خیال میں عملے کو دیکھ بھال فراہم کرنے کے دوران کن چیزوں کا خیال رکھنا چاہیے جس سے حاملہ خواتین کو محسوس ہو کہ ان کا عزت واحترام کے ساتھ علاج کیا جارہا ہے؟	
803	آپ کے خیال میں کون سا طریقہ سب سے بہترین ہے جس سے اس بات کی یقین دہانی کی جائے کو تمام خواتین کو عزت واحترام کے ساتھ دیکھ بھال فراہم کی جائے؟	
