# Supplementary material for: Health system bottlenecks hindering provision of supportive and dignified maternity care in public health facilities
Source: PLOS Glob Public Health. 2022 Jul 8;2(7):e0000550. doi: 10.1371/journal.pgph.0000550 (PMC10021678; doi:10.1371/journal.pgph.0000550)
Supplement: S3 Text — (DOCX) [file pgph.0000550.s003.docx]

**Operations of Obstetrics & Gynaecology Department in a secondary-level public health facility:**

A typical OBGYN unit of a public sector secondary care health facility runs in three shifts (morning, afternoon, and night). The team comprises of an obstetrician/gynaecologist (Section Head), supported by one or more doctors for each shift along with a nurse, midwife or lady health visitor in different combinations, and the standard non-clinical staff (*Aaya* or nursing aid, cleaner, and a ward assistant). Every staff member has defined roles and responsibilities; however, in sub-district health facilities the members of maternity teams go beyond these usual responsibilities to support each other. On-duty senior doctor takes clinical decision whereas administrative decisions of the section are taken by section in-charge. Doctors make the care plan for patients which is delivered by the nursing staff. In the absence of doctor, the senior nurse takes the decision while seeking guidance from the doctor over phone, as needed.

The head of Obs/Gyne department reports to the in-charge of a health facility who often visit entire health facility for monitoring purpose. The volume of births varies from 40 to 350 per month. Designated clinical staff members (on duty nurses) are responsible to fill service registers in the department. A clerk, reporting to facility in-charge is responsible to consolidate daily and monthly services report as per standard template. Monthly reports are sent to the district health office.
